# Modelling Infectious Hematopoietic Necrosis Virus Dispersion from Marine Salmon Farms in the Discovery Islands, British Columbia, Canada

**DOI:** 10.1371/journal.pone.0130951

**Published:** 2015-06-26

**Authors:** Michael G. G. Foreman, Ming Guo, Kyle A. Garver, Dario Stucchi, Peter Chandler, Di Wan, John Morrison, Darren Tuele

**Affiliations:** 1 Institute of Ocean Sciences, Fisheries and Oceans Canada, P.O. Box 6000, Sidney, B.C., V8L 4B2, Canada; 2 Pacific Biological Station, Fisheries and Oceans Canada, 3190 Hammond Bay Road, Nanaimo, B.C., V9T 6N7, Canada; 3 School of Earth and Ocean Sciences, University of Victoria, 3800 Finnerty Road, Victoria, B.C., V8P 5C2, Canada; Friedrich-Loeffler.Institut, GERMANY

## Abstract

Finite volume ocean circulation and particle tracking models are used to simulate water-borne transmission of infectious hematopoietic necrosis virus (IHNV) among Atlantic salmon (*Salmo salar*) farms in the Discovery Islands region of British Columbia, Canada. Historical simulations for April and July 2010 are carried out to demonstrate the seasonal impact of river discharge, wind, ultra-violet (UV) radiation, and heat flux conditions on near-surface currents, viral dispersion and survival. Numerical particles released from infected farm fish in accordance with IHNV shedding rates estimated through laboratory experiments are dispersed by model oceanic flows. Viral particles are inactivated by ambient UV radiation levels and by the natural microbial community at rates derived through laboratory studies. Viral concentration maps showing temporal and spatial changes are produced and combined with lab-determined minimum infectious dosages to estimate the infective connectivity among farms. Results demonstrate that neighbouring naïve farms can become exposed to IHNV via water-borne transport from an IHNV diseased farm, with a higher risk in April than July, and that many events in the sequence of farm outbreaks in 2001-2002 are consistent with higher risks in our farm connectivity matrix. Applications to other diseases, transfers between farmed and wild fish, and the effect of vaccinations are also discussed.

## Introduction

As aquaculture accounts for nearly fifty percent of the world’s food fish production, it has been cited as the fastest growing food-production sector of the world. The expansion of aquaculture, although disproportionate across countries, has occurred in almost all regions of the world amassing to a growth rate of nearly seven percent annually since 1970 (http://www.fao.org/fishery/topic/13540/en). However, concomitant with aquaculture intensification efforts has been a growing debate on the health [1], safety, and sustainability of the industry, and its impact on wild fish.

Canada, having the world’s longest coastline and some of the largest tidal ranges, is among the list of countries suited to meet the growing demand for fish through aquaculture production. Currently, farmed salmon dominates Canadian aquaculture production and accounts for approximately 85% (by volume and value) of the finfish cultured. Annually producing over 100,000 tonnes, Canada is among the top 4 global producers of farmed salmon. The majority of this production occurs in the coastal marine waters of British Columbia (BC) where over the past forty years the industry has evolved from rustic small individually owned pens to high tech multi-national corporations. Despite the dramatic improvements in farming techniques, BC farmed salmon production has remained relatively unchanged over the last decade due in large part to slow regulatory approvals of new sites stemming from public concerns that farming practices are damaging to wild stocks. One of the key issues in this debate is whether or not open net-pen salmon farms amplify and spread pathogens to the detriment of wild fish stocks. Although it is well accepted that most fish farm diseases come from wild hosts in the surrounding waters [2–4], there is less information available on the export and transfer of infections from BC fish farms to wild species. However, as there is increasing evidence elsewhere in the world that viruses may be transferred from farmed to wild fish through contact with discharges and products from infected farms [5], there is concern that it may also be happening in BC.

One of the most devastating pathogens of farmed salmon in BC is infectious hematopoietic necrosis virus (IHNV). The virus is endemic to the Pacific Northwest where it naturally infects Pacific salmon species that inhabit the waters shared with Atlantic salmon farms. Due to its efficient waterborne transmission and high virulence in Atlantic salmon, IHNV infections have spilled over from wild salmon to farmed Atlantic salmon in BC and Washington State. The ensuing virus outbreaks that occurred on the farms resulted in mortalities ranging from 18% to 78% and on multiple occasions resulted in the loss of tens of millions of dollars in revenues [6–8]. Additionally, because of the lack of familiarity with the transmissibility of IHNV in a novel host and the urgency of the biosecurity required to prevent its spread, IHNV became widespread amongst the industry during the initial outbreaks.

In a retrospective study investigating the spatial and temporal nature of IHNV outbreaks among 36 British Columbia salmon farms over the period of 2001 to 2003, it was determined that farming practices, such as boat movements and the use of shared personnel and contractors, had a significant contribution to the farm-to-farm spread of disease [7]. As a result, in 2010 the BC salmon farming industry implemented a viral management plan which enacted strict biosecurity measures to control the anthropogenic risk factors influencing viral spread (e.g., the immediate quarantine of an infected farm site) and they have proven effective at reducing the spread of disease. However as noted in [7], during the 2001–03 epidemic over half of the farms located 10 km or less downstream (based on expected estuarine dynamics) from an infected farm developed IHNV between 2 and 6 weeks following the outbreak in the upstream farm, suggesting that waterborne spread also likely played a role in spreading the disease.

**Fig 1:**
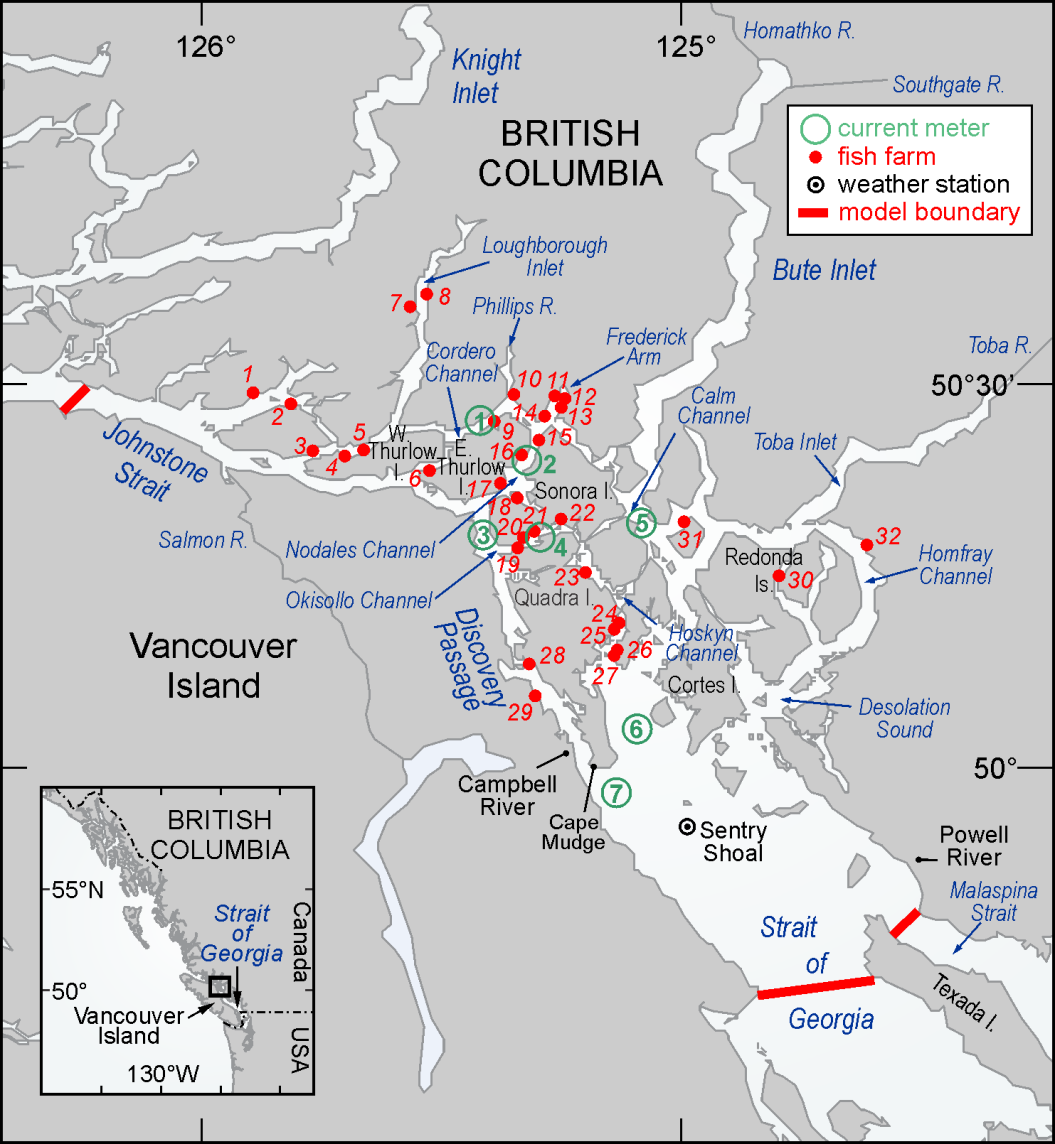
Map of the Discovery Islands region showing place names, rivers, salmon farms, current meter locations, the Sentry Shoal weather buoy, and the ocean circulation model boundary.

The Discovery Islands, situated between east-central Vancouver Island and the BC mainland (Fig 1), is one of the main farmed salmon producing regions of British Columbia. This area is characterized by a complex network of narrow channels and deep fjords that contain some of the strongest tidal currents in the world [9]. The extensive mixing of waters and close proximity of farms within this area may link the salmon farms epidemiologically. The recent development of an hydrodynamic ocean circulation model for the this region [10] and the publication of laboratory experiments that estimate waterborne transmission parameters associated with IHNV infection in Atlantic salmon [11] now make it feasible to quantify the risk of IHNV water-borne transmission in this salmon aquaculture intensive area. In the advent of an outbreak at a particular Discovery Island salmon farm, an appropriate summary of these simulations could provide industry with an indication of where the virus might disperse, the associated waterborne viral concentrations, and estimates of the likelihood that nearby farms will become infected.

Similar modelling studies have been carried out in other salmon farming regions of the world and a comprehensive review of the role that hydrodynamic and other models (e.g., epidemiological) can play (and have played) in estimating the transmission of disease-causing agents from open-net farms was recently presented in [12]. Though there have been a wide range of hydrodynamic models employed, the approaches are generally similar and the particular applications are simply a reflection of the need to capture the unique flow and water properties in each of the of the major salmon farming regions.

Norwegian modelling studies (e.g., [13–15]) have generally focussed on transmission of the salmon pancreas disease virus and salmon lice (*Lepeophtheirus salmonis*) from farms that are primarily located in coastal inlets and fiords similar to those in BC. Their modelling has addressed issues like seasonal differences in the freshwater runoff from the surrounding mountains and the need for accurate atmospheric and outer boundary forcing, each of which are important for BC hydrodynamic models. Chilean salmon farms are located in similar regions and thus modelling studies [16–17] aimed at better understanding the large outbreak of infectious salmon anaemia (ISA) which began in 2007 and spread to seventy-six farms over a fifteen month period, are also relevant. Scottish salmon farms are typically in lochs, and though their surrounding terrain is not as mountainous as in Norway, Chile and BC, the analyses they employ for studying both sea lice [18–21] and disease transmission [22–23] issues are also applicable. For example, [21] is particularly notable in its use of model results to calculate dispersion probabilities and a connectivity matrix to identify farms that should be critical in controlling the overall loch population of sea lice. Disease and sea lice are also problematic among the salmon farms southwest New Brunswick and northeast Maine and several relevant modelling studies have been conducted [24–27]. However their ocean circulation is strongly dominated by the M_2_ tidal constituent and as a result, the wind and river discharge forcing fields that have comparable importance to tides in other parts of the world are often ignored in their pathogen dispersion simulations. Consequently, even though the hydrodynamic and particle tracking models used in that region are very close to those employed here, their widespread use of M_2_ tidal excursions as a basis for estimating dispersion and management zones is not relevant to BC. However, hydrodynamic and particle tracking models very similar to those that will be described here, were developed and used to simulate the dispersion of sea lice from salmon farms in the Broughton Archipelago [28]. This region is just to the northwest of the Discovery Islands and has similar topography, waterways, and many of the same forcing issues. So that modelling study is highly relevant to what is described here. In particular, the Broughton circulation model was forced with tides, winds and freshwater discharge and evaluated against acoustic Doppler current profiler (ADCP), tide gauge, and salinity/temperature observations, analogous to what was presented for the Discovery circulation model in [10].

## Materials and Methods

### Ocean Circulation Model Overview

Currents in the Discovery Islands region (Fig 1) arise from a combination of tidal, atmospheric, and freshwater discharge forcing and, as illustrated in [10], have considerable spatial and temporal variability. Though observations at specific locations have provided an indication of these variations, the costs of deploying, maintaining, and recovering current meter moorings are sufficiently prohibitive that it is unlikely there will ever be adequate coverage to produce the spatially-detailed fields needed to carry-out accurate viral transmission studies among salmon farms in the region. As done in other regions (and discussed in the introduction), an alternative strategy is to develop a numerical ocean circulation model with sufficient accuracy and spatial resolution that it can provide these currents, as well as associated sea surface elevations and three-dimensional (3D) salinity and temperature fields, over specific time periods. The development and accuracy evaluation of such a circulation model for the Discovery Islands region was described in [10] and the Appendix provides further details on the specific forcing fields, initial and boundary conditions, and model simulations (and their evaluation) that generated the current fields employed here. In particular, these fields were output and stored hourly for the entire model domain for the months of April and July 2010 and then subsequently input to the particle tracking program. Each of these monthly files is approximately 20 gigabytes.

### The IHNV Dispersion, Inactivation, and Re-infection Model

The IHNV particle tracking approach used in the Discovery Islands model is similar to that employed for sea lice in the Broughton Archipelago and described in [28]; it just incorporates different biology. In this case, the relevant parameters or relationships are i) the viral shedding rate from an infected Atlantic salmon farm, ii) the viral survival rate in the marine environment, and iii) the minimum infective dose required to infect naïve Atlantic salmon. These parameters were estimated by a series of laboratory and field experiments whose methodologies and results were described in [11]. In seawater, the inactivation of IHNV was found to be largely determined by exposure to sunlight (UV A and B radiation) and by the natural microbial community (e.g., bacteria). As the hydrodynamic circulation model does not compute UV radiation, it was estimated from UV measurements (A and B bands) collected by a Davis Instruments UV erythemal sensor at many of the Discovery weather stations, adjusted to account for UVA attenuation [11], and then reduced by 8%, the average ocean surface albedo for UV radiation. Time series of the resultant sea surface UV radiation values for April and July 2010 are shown in the Appendix.

The exponential decay, exp (-α z), of UV (A and B) radiation as a function of water depth z below the surface was determined by fitting a curve to several measurements taken with a multichannel UV radiometer at a few Discovery locations over two days in early September 2010. Although the coefficient α was estimated to be 0.6396 m^-1^, the results are expected to be highly dependent on particulate material (e.g., sediment, phytoplankton) in the water column. Thus the coefficient probably varies in both space and time and this will introduce uncertainties to our subsequent estimates of virus survival in the marine environment. The exponential decay rate of IHNV caused by the natural microbial community (exp(-β t) where β = 4.18 day^-1^, and t is the time in days) was determined from experiments conducted using water collected from the Strait of Georgia near the Pacific Biological Station (PBS) over a short time period [11]. This rate of viral dispersal may also vary in both time and space, contributing to further uncertainty. Tests that explore the sensitivity of model results to one of these parameters are described in the last section.

The viral transport simulations were performed through the release of thirty virus particles per hour from each of the thirty-two farms shown in Fig 1. (The value of thirty was chosen as a compromise between the need for estimating diffusion within the flow fields and the computer time required to carry-out the actual particle tracking.) These particles were subsequently tracked for a period of eight days, with their positions stored every 20 minutes, to encompass the viral infectious time period as empirically determined in [11]. The releases were randomly spaced over a 100m by 100m by 20m volume that approximately represents the dimensions of a typical farm site. In the virus simulations, each particle represented a cohort of infectious viruses (denoted as plaque forming units—pfus) whose precise number is scaled-up later to be consistent with diseased fish shedding rates that were determined in laboratory experiments [11]. The viral particles were dispersed by the model currents and inactivated by exposure to UV and the background microbial community in the water. The cumulative inactivation was computed as the time integral of exposure over hourly time periods (the frequency of the UV observations), as described in [11].

A key question is whether viral concentrations arising from a disease outbreak at one farm are sufficient to trigger an outbreak at a neighbouring farm. The answer requires invoking the minimum infective dose of 10 pfu ml^-1^ (= 10^7^ pfu m^-3^) that [11] found for Atlantic salmon and further scaling our numerical particle releases to represent worst case and more realistic scenarios. (Note that 10 pfu ml^-1^ was the actual concentration of virus present in the sea water tank when Atlantic salmon were subjected to the virus trials described in [11]. This concentration was determined by cell culture plaque assay and did take into account the inoculum dilution that was plated when determining the minimum virus concentration in one milliliter of sea water required to trigger an IHN disease event in Atlantic salmon.)

The worst case scenario number was estimated as follows. The average peak shedding rate of IHNV infected Atlantic salmon is 3.2 ×10^7^ pfu/fish/hour, a rate obtained from fish in the terminal stages of IHN disease, and that represents the maximum level of virus shedding that occurs during the course of infection. Consequently, during an outbreak on a farm not all infected fish will be at the same stage of illness whereby they are all shedding at a maximum level. However for the purposes of modelling, if we assign the peak rate to all infected fish in a pen then we will be confident that the number of virus particles being released will truly represent a worst case scenario. Another consideration when determining the number of virus particles shed from a farm is the number of fish that become infected and diseased during an outbreak. As in any population, not all fish on a farm will develop IHN disease during an outbreak. By utilizing the number of mortalities during past outbreaks as a proxy for determining the number of fish that developed IHN disease and shed virus, we deduce that 1% of the farm population per day would represent a worst case scenario. (It is assumed that the 1% mortalities are removed and replaced by virus shedding fish that have progressed to that peak shedding stage.) So if a typical farm is assumed to have 500,000 fish on site and the 1% that are infected are shedding at the peak rate, then 1.6 × 10^11^ pfu will be released per hour from that farm site.

However due to the development of rapid detection methods, the use of vaccinations, and regulations requiring the immediate removal of infected populations, the occurrence of such IHNV worst case scenarios have been made nonexistent. Therefore to simulate a more realistic scenario, the risk of virus transport was also evaluated in the context of a vaccinated salmon farm. Since the Canadian licensure of the DNA vaccine, Apex-IHN, in 2005 over 60 million doses have been administered among BC salmon farms and have proven to be highly efficacious against IHN disease in Atlantic salmon ([29]; Garth Traxler, personal communication). Consequently, farms in the Discovery Islands region that vaccinate their fish against IHNV have both a lower risk of becoming infected and as a population and a greatly reduced capacity to shed virus. In vaccine efficacy trials conducted at the Pacific Biological Station it was found that out of one hundred Apex-IHN vaccinated Atlantic salmon only two fish developed IHN disease and shed virus after being exposed to a lethal dose of virus (Garver et al., manuscript in preparation). Based on these results, we then estimate that if an IHNV vaccinated salmon farm of 500,000 fish were subjected to a lethal dose of virus, approximately 2% would be susceptible to infection. Thus reducing the number of IHNV susceptible fish to 10,000 and assuming both a1% disease prevalence and an IHNV disease progression that is the same within a vaccinated population as it is in an unvaccinated population, virus shedding from the vaccinated population would at most reach a rate of 3.2× 10^9^ pfu/hour per farm.

Using the preceding assumed values for farm dimensions and number of fish per farm, the average density is 2.5 fish m^-3^. In order to infect an otherwise naïve farm, only 1 fish need become infected and so we only need a viral cohort to enter into the three-dimensional (3D) region occupied by a farm and spend at least one hour there (the same time that naive fish were exposed to pfus in the laboratory experiments [11]). As each cohort represents (1.6 × 10^11^)/30 pfus, its fractional survival (1.–inactivation) over the period it is within the naïve farm need only be (10^7^)/ ((1.6 × 10^11^)/30) = 1.9 × 10^−3^ for the pfu concentration in a 1 m^3^ volume around it to exceed the minimum infective dose. Therefore to determine if an infection from farm A spreads to farm B, we need only determine if at some time in its trajectory, a cohort shed from A enters within the 3D domain of B and has fractional survival greater than 1.9 × 10^−3^. (When the shedding farm is vaccinated, this value rises to 0.095.) The risk of infection from A to B can then be quantified as the percent of total shedding time from A for which there is at least one cohort within farm B whose fractional survival exceeds the minimum threshold.

## Results

### April and July 2010

**Fig 2:**
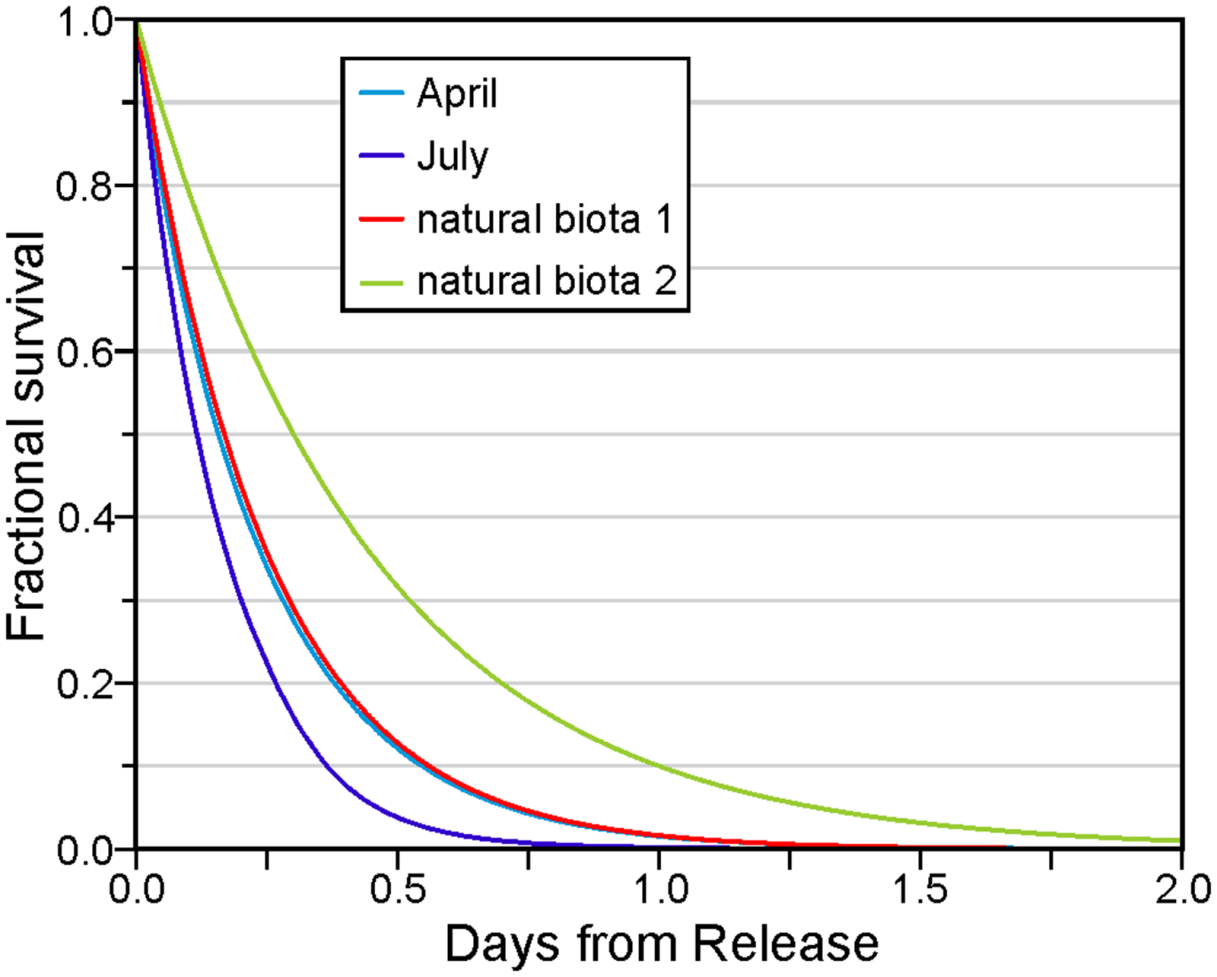
Average fractional IHNV survival as a function of time from release: April (light blue) and July (purple) curves are due to both UV and natural biota, while biota 1 (red) and biota 2 (green) arise from only the natural biotic decay coefficients of 0.418 day^-1^ [11] and 2.30 day^-1^ (discussed in Summary).

Consistent with the UV levels shown in the Appendix, Fig 2 shows that the fractional survival of viral cohorts, averaged over all releases from all farms, was lower in July than April. The respective April and July average values after one day were 0.016 and 0.002, and the largest value after eight days for all cohorts was 6 × 10^−14^. However as the April survival curve is only slightly lower than that arising from natural biota with the decay rate estimated from [11], UV must be playing a very minor role. It clearly has a larger impact in July. Though this relatively fast average inactivation would seem to suggest that our tracking could be curtailed much sooner than eight days, this time period is necessary for the sensitivity test described in the final section. And though the details are not shown here, consistent with the average and rms currents listed in the Appendix, the average (over all releases from all farms) particle distance travelled over eight days was about 10% greater in July than April, and the dispersion (computed as the standard deviation of these distances) was 31% larger.

**Fig 3:**
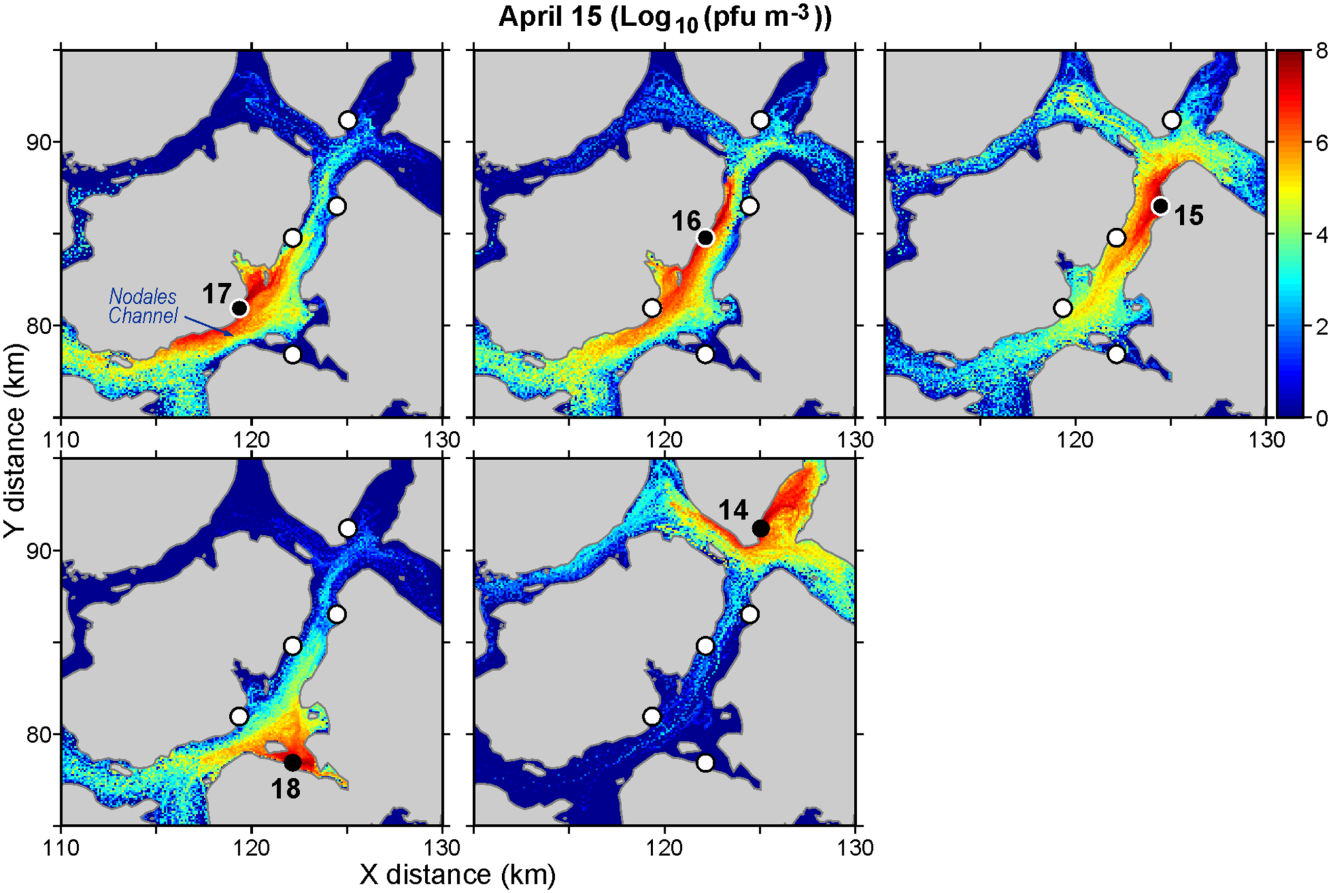
Average virus concentrations (log_10_ (pfu m^-3^)) over the top twenty meters of the water column on April 15, 2010 arising from releases of 1.6 × 10^11^ pfu/hour at five farms around Nodales Channel. Black circles denote the release (diseased) farm (numbered as in Fig 1) while white circles show locations of the other four.

**Fig 4:**
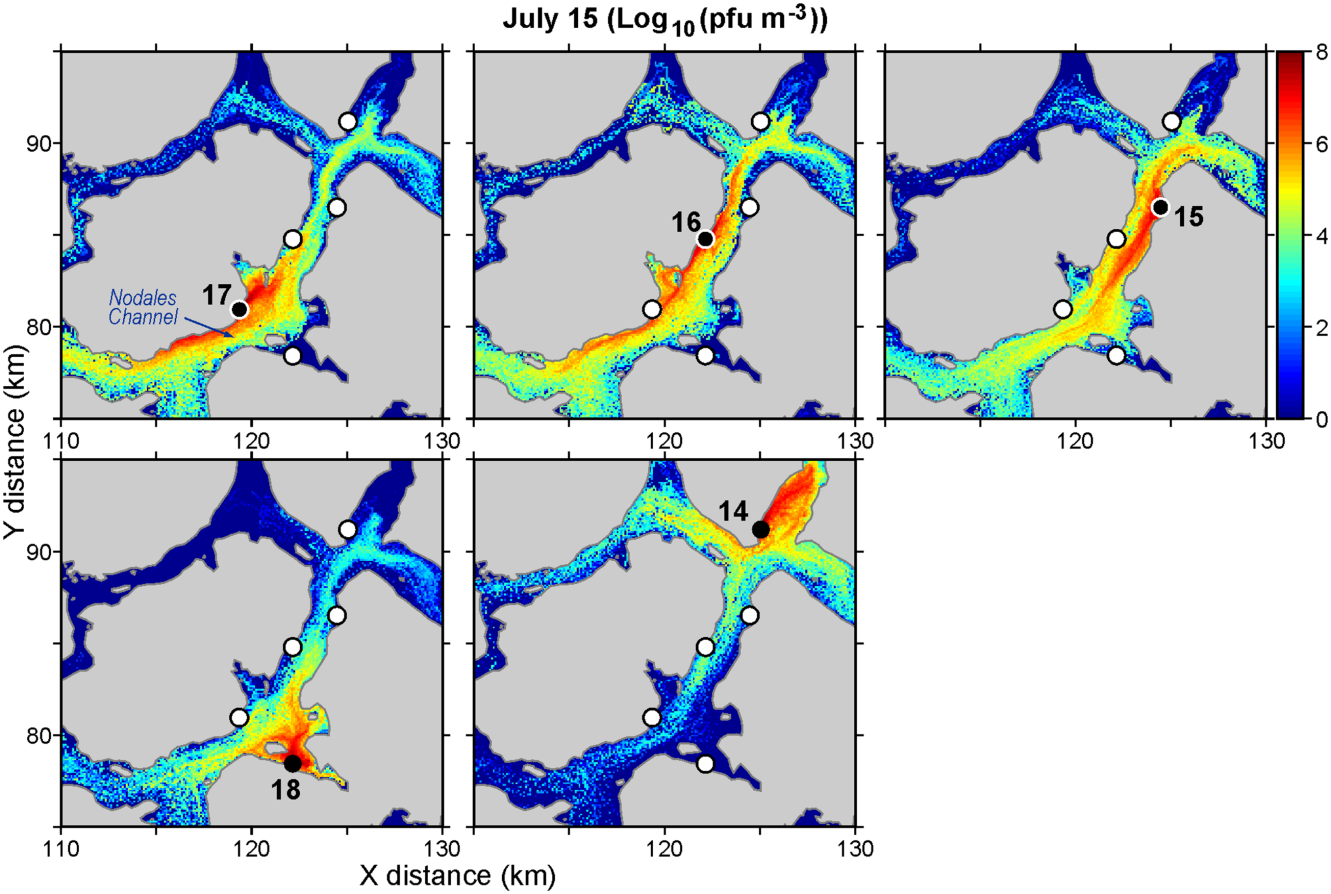
As in Fig 3 but for July 15.

As an example of the output from these particle tracking studies, Figs 3 and 4 show daily average virus concentrations (pfu m^-3^) over the top 20m of the water column for April 15 and July 15, respectively. (The concentrations were computed by summing the proportion of active cohorts in volumes of size 100m by 100m by 20m covering the model domain.) Only releases from five farms around Nodales Channel (Fig 1) are considered and they have been scaled-up to represent a shedding rate of 1.6 × 10^11^ pfu/hour/farm. Though not shown, it should be noted that these concentrations do vary over a twenty-four hour period as the exposure to UV radiation varies. Specifically, concentrations in the evening after exposure to UV radiation over the preceding daylight hours are smaller than those around sunrise that have gone without exposure the preceding night.

The average concentration over the sub-region shown in Fig 3 is 6.1 × 10^9^ pfu/m^3^ while that for Fig 4 is 5.3 × 10^9^ pfu/m^3^. So there are more virus on April 15 than on July 15. Both dates are within two days of maximal spring tide conditions [9], so the tidal dispersion leading up to these dates should be similar. Furthermore, observations at the Lee Island weather station (located close to farm 16) indicate that wind was not a factor on either day; the average hourly speeds on April 15 and July 15 were 0.8 and 0.5 m s^-1^, respectively. Thus most of the difference should be attributable to July having both larger UV values that would lead to higher mortality rates, and larger near-surface currents that would tend to transport and disperse the viruses further afield. (See Appendix for details.)

**Fig 5:**
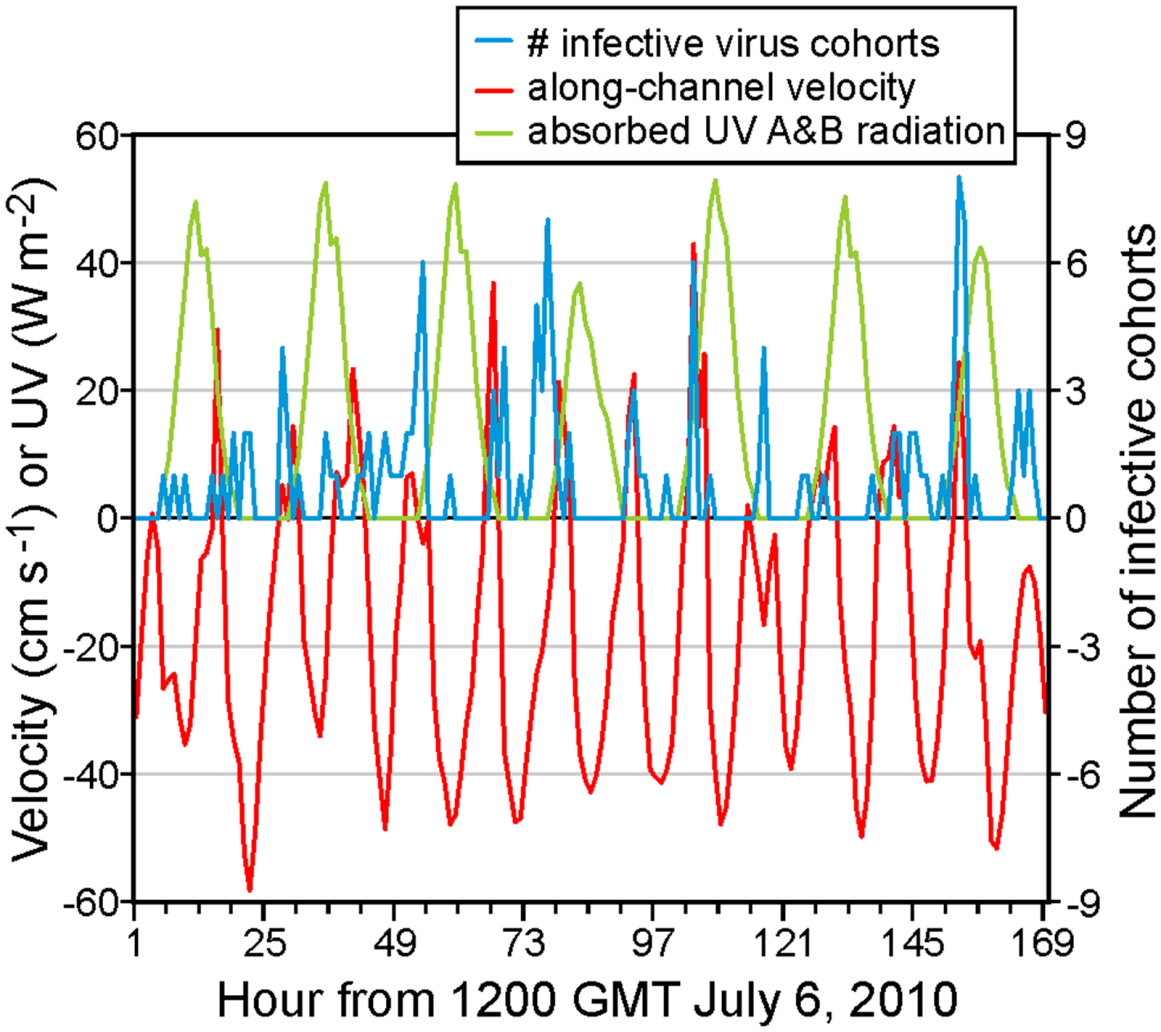
Number of infective cohorts arriving at farm 17 from farm 16 (blue), along-channel model velocity (cm s^-1^) at 10 m depth at the location of mooring NC1 (red), and UV (A and B) radiation (W m^-2^, green), as in Fig 4. The time period is 1300 GMT July 6 2010 to 1300 GMT July 13 2010.

Fig 5 illustrates the relationships between the number of infective cohorts arriving at a naïve farm, the model velocities, and the UV (A and B) radiation. The releasing (pseudo-diseased) farm is 16 and the receiving (pseudo-naïve) farm is 17, both in Nodales Channel (Fig 1). The velocities have been taken at the location of ADCP mooring NC1 (#2 in Fig 1) at 10m depth and have been resolved into their along-channel component, with positive values denoting flows to the northeast. They are clearly seen to be comprised of oscillating tides and a mean southeastward flow of approximately 12 cm s^-1^. The time period is 1300 GMT July 6 to 1300 GMT July 13, the number of arriving infective cohorts has been lumped into hourly segments, and the UV values are as in shown in the Appendix. For the most part, it can be seen that infective cohorts arrive at farm 17 when the flow is to the southeast (i.e., negative) and the UV radiation (hence inactivation) is small. However as expected from Fig 2, the infective cohort relationship with UV is much weaker in April (not shown). And at other locations, such as between farms 4 and 5 where a stronger surface estuarine flow means that the along-channel velocities are seldom eastward, the relationship between the number of arriving infective cohorts and the along-channel velocity is less clear.

**Fig 6:**
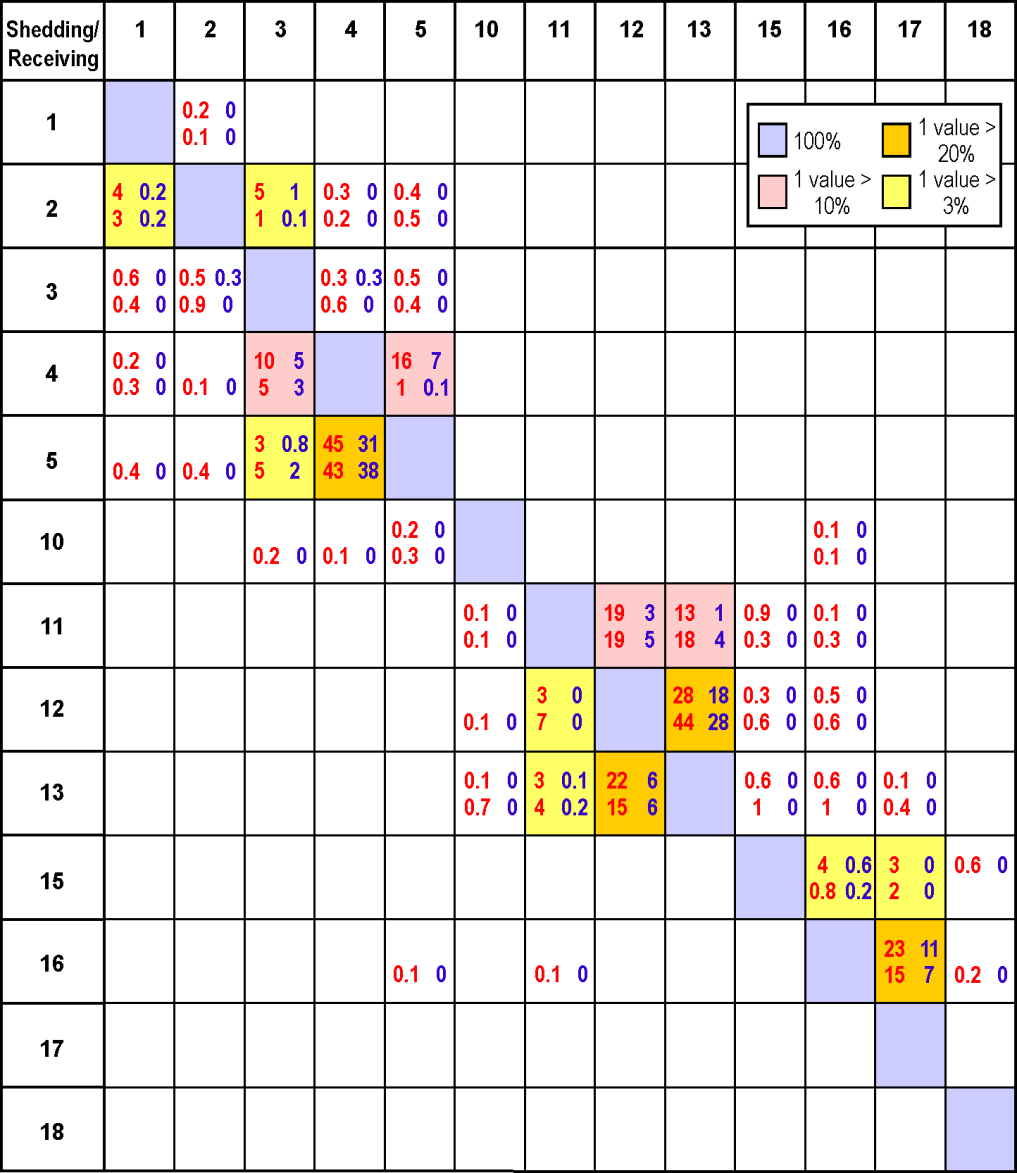
Percentage of hours when the viral concentrations arising from worst case (red) and vaccinated (blue) scenario disease outbreaks at (shedding) farms in April (upper line) and July (lower line) exceed minimum infective dose thresholds at nearby (receiving) unvaccinated farms. See Fig 1 for farm locations. Shedding farms are along the y-axis and receiving farms along the x-axis. Blanks denote zero, decimal places are only shown for nonzero values less than 1, and diagonal (100%) values are shaded in light blue. Boxes with at least one value larger than 20%, 10% and 3% are shaded in orange, yellow, and pink, respectively.

Fig 5 is only presented for illustrative purposes. Similar figures could be produced for other time periods in April and July and all possible combinations of release and receiving farms. The relative risk of water borne transmission between diseased and neighbouring naïve farms can then be quantified by placing average infective cohort counts, like those displayed in Fig 5, into a connectivity table similar to those produced in Table 1 of [13] and Table 2 of [14]. As an example, Fig 6 shows April and July connections risks for the same 13 Discovery farms that were presented in [7]. (Full tables showing connections among all 32 Discovery farms will be generated and made available to industry and FOC Aquaculture Management Division.) In this case, the metric chosen to denote connectivity is the percentage of time over days 6–20 of each month when the concentration in at least one m^3^ volume within the receiving farm exceeds the minimum infective doses arising from a worst case scenario or vaccinated shedding farm. (For now, the receiving farm is assumed to be unvaccinated but the further risk reduction when it is also vaccinated will be discussed later.) This calculation assumes continuing hourly releases (i.e., no measures were taken to stop the viral shedding) and the 15 day period (as opposed to the full 18-day) was chosen to account for a full spring-neap tidal cycle and for spin-up and spin-down periods when the concentrations were not at relatively steady values.

In Fig 6 it is important to notice that even though the IHNV vaccination is 98% effective, the percentages arising from a disease outbreak at a vaccinated farm are in many cases, greater than 2% of those that would arise if that farm were not vaccinated. In other words, if these percentages are interpreted as risks, the risk does not decrease proportionately with the effectiveness of the vaccine. Though it is true that the viral shedding and concentrations from a vaccinated farm will be 2% of those from the same farm without vaccination, because we are not taking into account the amount by which a particular concentration exceeds the threshold, only whether or not it is exceeded, our risk function is nonlinear. So for example, if the viral concentration arising from an unvaccinated farm were 10^*9*^ pfu m^*-3*^, thereby exceeding the minimum infective dose by a factor of 100, the concentration that would arise if the shedding farm were vaccinated would be 2% of that value, which still exceeds the minimum infective dose by a factor of 2. So our connectivity risk metric would count both cases equally. This certainly seems to be what has happened for transmission from farm 5 to farm 4 where Fig 6 shows that vaccination has only reduced the April risk from 45% to 31%. In the absence of laboratory experiments with vaccinated fish, it is difficult to extend these results to the case when the receiving farm is also vaccinated. However, the viral concentrations in the water would be the same but only 2% of the population on the receiving farm would be susceptible to infection. Consequently due to the reduction of susceptible farm fish, the likelihood of the initiation and spread of IHNV disease would be substantially reduced.

Due to the higher UV values and more dispersion, the July values in Fig 6 are generally lower than those for April. Notable exceptions are for farm 5 transmitting to farms 3 and 4, and farms 11, 12, and 13 whose exchanges among themselves are presumably larger due to currents arising from the nearby larger freshwater discharge at the head of Frederick Arm.

### Application to the 2001–02 Disease Outbreaks

Despite the obvious timing mismatch, it is interesting to compare the relative risks in Fig 6 with the sequence of 2001–02 Discovery IHNV outbreaks reported in [7]. Our simulations and analyses are only for April and July 2010 while those discussed in the area 1 (Discovery) outbreaks in [7] span 225 days, beginning “late August 2001”. Even if we assume “late” means August 31, day 225 would be April 13, 2002 which only has minimal overlap with our April particle tracking which began on April 6. Furthermore, we only released viruses over 18 days each month whereas the epidemic duration on each farm (Fig 2 in [7]) typically spanned three months. Nevertheless our connectivity matrix and flow fields may shed some light on the role that water-borne transmission may have played in the sequence of outbreaks.

**Fig 7:**
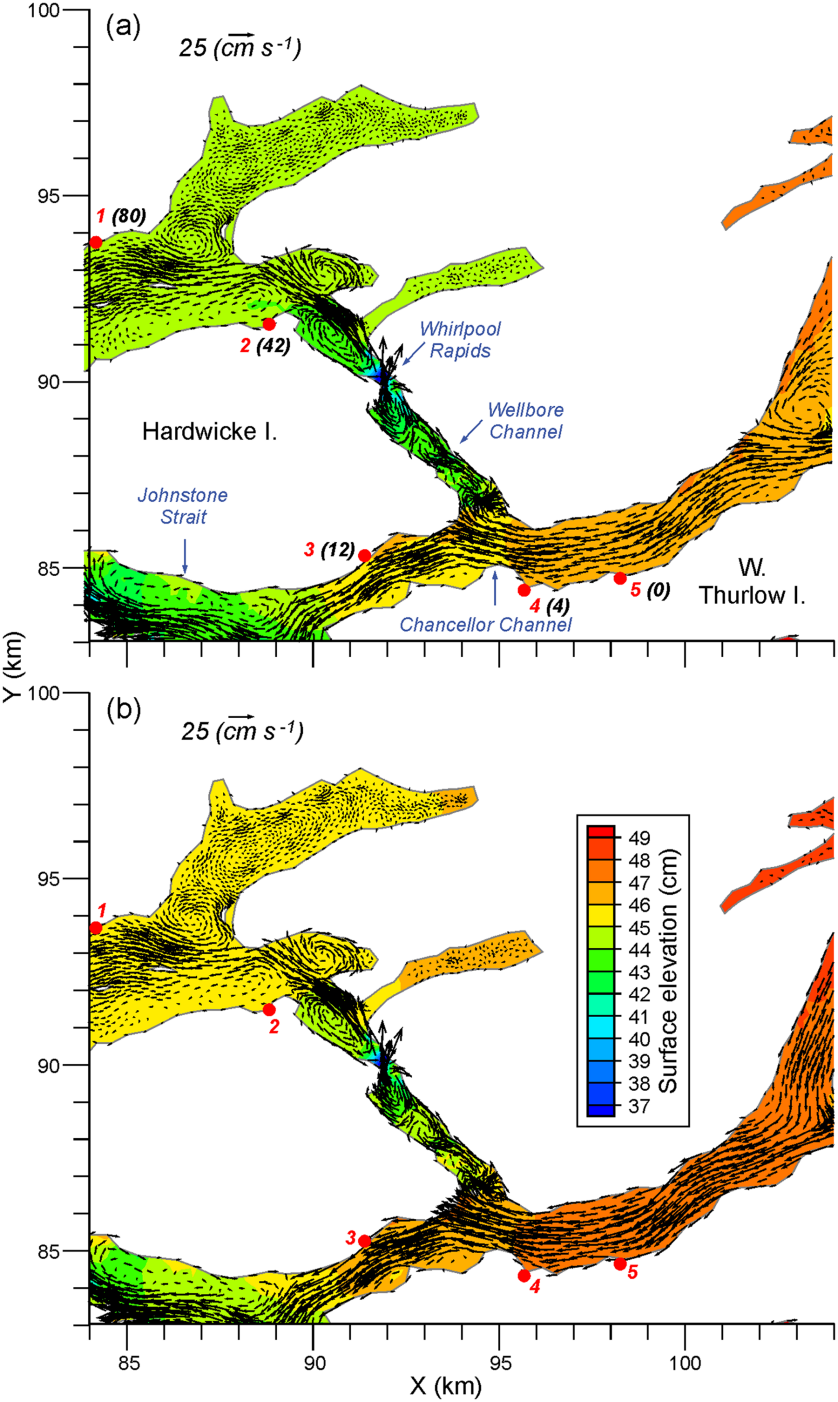
Average a) April and b) July mean surface elevations (cm) and flows (cm/s) at 10m depth in the Chancellor Channel region. Numbered red dots denote farms, as in Fig 1 while the black bracketed numbers denote the number of days since the first IHNV outbreak was reported at farm 5 in August 2001. (These numbers were taken from Table 1 in [7].)

The first disease diagnosis was at the farm which is labelled 5 in Fig 1 ([7] numbered it as farm 1). Within 4, 12, 42, and 80 days, outbreaks were diagnosed at farms 4, 3, 2, and 1, respectively, again as numbered on our Fig 1. As suggested by Fig 2 in [7], these diagnosis times do not necessarily coincide with the time when the outbreak (“epidemic”) began. Nor, from the perspective of our modelling, do they correspond to the times when the minimum infective dose was first exceeded. However as discussed in [7] and seen in Fig 7, the sequence of outbreaks is consistent with a downstream progression that arises from the near surface estuarine flow. The relatively rapid transmission from farm 5 to farms 4 and 3 is certainly consistent with the April and July mean westward flow fields shown in Fig 7 and the relatively large connectivity values linking farms 5, 4, and 3. Furthermore, the considerably longer times required for the infections to reach farms 2 and 1 is consistent with both the more complicated mean flows (e.g., numerous eddies) that are shown in Wellbore Channel, the constriction at Whirlpool Rapids, and the lower connectivity table values. Note that in both April and July, farms 5, 4, and 3 are only weakly connected to farms 2 and 1. If water-borne transmission were the infection vector, according to Fig 6 the outbreak at farm 2 would most likely have come from farm 3 while the outbreak at farm 1 would have come from farm 2. This sequence is certainly consistent with the flow fields shown in Fig 7.

**Fig 8:**
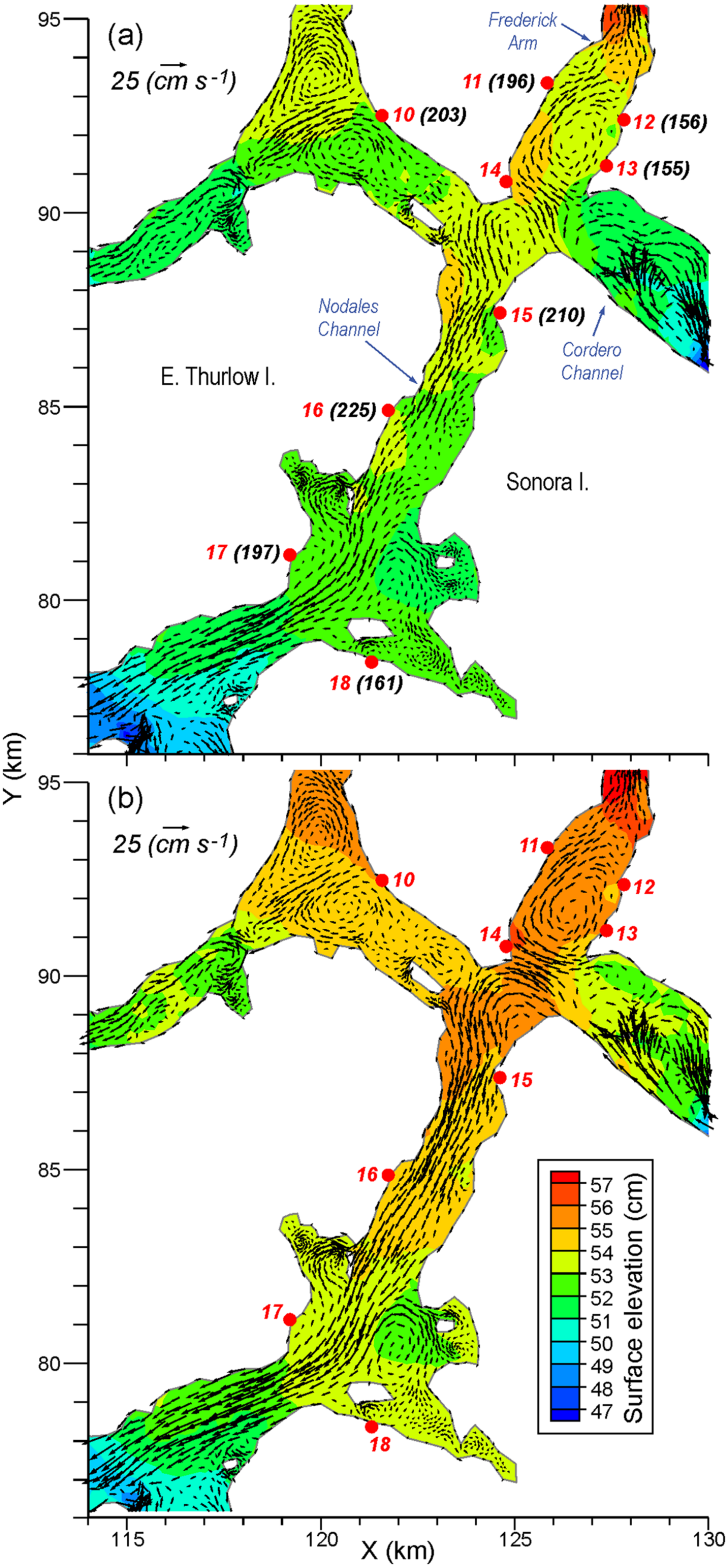
Average a) April and b) July mean surface elevations (cm) and flows (cm/s) at 10m depth in the Nodales Channel region. Numbered red dots denote farms, as in Fig 1, while the black bracketed numbers denote the number of days since the first IHNV outbreak was diagnosed at farm 5 in August 2001. (These numbers were taken from Table 1 in [7].)

Approximately 2.5 months after these five IHNV outbreaks, further outbreaks occurred in the Frederick Arm and Nodales Channel region (Fig 1). As the mean flow fields shown in Fig 7 are generally westward, it is unlikely this second outbreak was caused by water-borne transmission from the previous five infected farms. This is substantiated by Fig 6 which does not show any connection from farms 1–5 to the others. The sequence of outbreaks for this second set is shown in Fig 8a, along with the monthly-average 10 m flow fields for April 2010. The initial diagnosis was at farm 13 and the infection then spread within 1 and 6 days to farms 12 and 18, respectively. The former is only 1 km away and though [7] notes its location to be upstream from farm 13, our average flow field shows a clockwise eddy that, in addition to the oscillating tidal currents, could easily connect the two farms. The relatively strong connection from 13 to 12 in Fig 6 substantiates this claim. As noted in [7] and seen in Fig 8, farm 18 is about 10 km downstream so water-borne transmission within 6 days is certainly feasible. But the relatively rapid virus inactivation with time (Fig 2) decreases the likelihood and Fig 6 does not show such a connection. In fact, as Fig 6 does not show any direct connection from either farm 12 or 13 to farm 18, it is much more likely that this outbreak was a result of poor biosecurity or from virus spill-over from a wild source, rather than water-borne transmission from an infected farm. 41, 42, 48, 55, and 70 days after the outbreak at farm 13, the next farms to be infected were 11, 17, 10, 15, and 16, respectively. This sequence of outbreaks is not consistent with the mean flow fields seen in Fig 8, though again as depicted in Fig 2 of [7], diagnosis of the disease does not necessarily correspond with the beginning of the epidemic. Fig 6 does show that i) farm 11 could be infected from farms 12 or 13, ii) 17 could be infected from 13, 15, or 16, iii) 10 and 15 have only weak connections to 11, 12, and 13, and iv) 16 has only stronger connections to 15. So although water-borne transmission may have played a minor role, poor biosecurity practices among workers moving between the farms or naturally infected wild fish are more likely transmission agents [7]. Nevertheless, as indicated in Fig 6, water-borne transmission cannot be discounted completely.

Given the relatively fast average inactivation shown in Fig 2, it can expected that the minimum transmission time between farms that show relatively strong connections in Fig 6 is short. And indeed, that is the case. For example, the minimum time between shedding from farm 5 and arrival at farm 4 is 1.7 hours, while analogous times for 11 to 12, 11 to 13, 12 to 13, 13 to 11, and 16 to 17 are 4.3, 6.3, 1.0, 8.3, and 6 hours, respectively. As shown in Fig 5, in many cases these values arise when the currents and UV conditions are most favourable; i.e., when the tidal currents are in the correct direction and at night when UV is zero. Nevertheless, if a disease outbreak were to arise at farms 5, 11, 12, 13, or 16, and left to progress to mortality rates reaching 1% or greater, then it is likely that neighbouring farms would be exposed to shed virus, albeit perhaps not with a sufficient concentration to exceed the minimum infective dose.

## Summary, Discussion and Future Work

The preceding presentation has described the application of finite volume ocean circulation and particle tracking models to estimate the water-borne dispersion of the IHN virus among thirty-two Atlantic salmon farms in the Discovery Islands region of British Columbia. In order to demonstrate the impact of seasonal variability in the flow fields and UV radiation levels, historical simulations were carried out for April and July 2010. Numerical particles were released from infected farms in accordance with shedding rates estimated in [11] and inactivated by ambient UV radiation levels and the natural microbial community at rates that were also described in [11]. Viral concentration maps and plots showing temporal and spatial changes were produced and combined with lab-determined [11] minimum infectious dosages to estimate infection connectivity among farms. Results demonstrated that neighbouring naïve farms can become infected via water-borne transmission, with a higher risk in April than July, and that some of the sequence of outbreaks observed in 2001–2002 were consistent with values in our connectivity matrix. The other outbreaks could be a result of virus transmission from a wild reservoir or as suggested in [7], were likely due to poor farming practices.

These model results certainly support an adherence to the stringent disease management practices (e.g., the immediate quarantine of an infected farm site) that were invoked in 2010. If epidemics on diseased farms are not addressed quickly (which Fig 2 in [7] suggests was the case in 2001–02) then the risk that diseased farms will transmit infectious doses to other farms, and presumably to wild fish, becomes higher. However, our model results suggest that if action is not taken to remove an infected population before it reaches the 1% daily mortality rate, viral transmission to neighbouring naïve farms can happen and minimum infective doses can be exceeded within a few hours.

Another strategy for reducing the risk of disease outbreaks is through the use of commercially available IHN vaccines. Our model simulations demonstrate that this strategy greatly reduces the risk of transmission by lowering the overall number of susceptible fish and hence the virus shedding capacity of the vaccinated farm population if it were actually to become infected with IHNV. It is worthwhile noting that since the initiation of the use of the APEX IHNV vaccine in 2005, there has been no occurrence of virus in vaccinated populations while unvaccinated populations have been declared IHNV positive, further corroborating that this strategy reduces the risk of IHNV transmission. Nonetheless, in an effort to contrast virus dispersion from an unvaccinated farm versus from a vaccinated one, we simulated an IHNV outbreak in a vaccinated farm population using a 1% mortality rate among the susceptible fish (as was done for an unvaccinated farm). However by assigning an equivalent rate as observed to an unvaccinated population, we are likely inflating the amount of shed virus in this population as the disease severity and transmission would be greatly abbreviated in a population with far fewer susceptible individuals. Given these caveats, it is shown that for our choice of risk metric the reduction does not decrease proportionately with the effectiveness of the vaccine. For example, it was shown that the risk of an April outbreak at farm 5 spreading to farm 4 is only reduced by about 31% when farm 5 is vaccinated, as opposed to when it is not. (For both these cases, it is assumed that receiving farm is not vaccinated.)

Though this study focussed on farm-to-farm transfers, with a few modifications the same modelling tools could equally well be applied to other diseases and to transmission between farmed and wild populations, and vice versa. Of course, if circulation models exist or are developed, similar studies could be carried out in other regions. Farmed-to-wild transmission is a topical issue in BC as recommendation 19 of [30] stated that “on September 30, 2020, the minister of fisheries and oceans should prohibit net-pen salmon farming in the Discovery Islands unless he or she is satisfied that such farms pose at most a minimal risk of serious harm to the health of migrating Fraser River sockeye salmon.” Laboratory work has begun to investigate the susceptibility of migrating sockeye salmon to IHN disease and field work is continuing to better understand the behaviour, timing, and particular routes taken by these fish on both their outward migration through the Discovery Islands as juveniles, and their inward migration as adults. Once these are better understood, it will be possible to carry-out simulation scenarios that estimate the risk of infection for sockeye swimming in proximity to diseased farms and assess the relative effectiveness of disease and farm management practices such as vaccination, quarantine, depopulation, and farm siting changes.

Equally important to differentiating whether or not farm sites are point sources of virus is an understanding how and when farms may become infected with IHNV. Although the marine source of virus for farmed salmon has not been conclusively defined, IHNV is commonly present in many Pacific salmon species [31] that naturally share the marine waters with open net-pen Atlantic salmon farms. Recent laboratory studies have revealed that sockeye salmon smolts can carry IHNV and depending on the particular stock, may be asymptomatic carriers. Additionally, laboratory studies have demonstrated that IHNV infected sockeye salmon are capable of transmitting virus to naïve Atlantic salmon when they are in close proximity [32], further suggesting that sockeye salmon may be a potential source of virus to farmed fish. With a better understanding of migratory behaviour and IHNV prevalence in sockeye salmon stocks, it should be possible to simulate wild-to-farm disease transmission events and develop appropriate predictive tools.

There are numerous assumptions and/or limited observations associated with the preceding results and it is worthwhile reviewing them here. The number of infected fish shedding virus from an unvaccinated farm was assumed to be 5000 per day, 1% of a total population of 500,000. That number is probably too high for current farm management practices but it may not have been for the practices that were in place during the outbreaks in 2001–02. Our UV extinction rate with depth was based on limited measurements at a few locations and certainly could vary both seasonally and spatially (e.g., with different sediment and phytoplankton loads in the water column) throughout the Discovery region. Likewise, the amount of background biotic material in the water and its impact on viral inactivation was based on limited observations and undoubtedly also varies in both space and time. And though migrating or pelagic stocks can play a role transmitting disease to farms [2] and thus may have confounded the outbreak analysis described in [7], those effects have not been considered in the model simulations described here. With increasing observations on the distribution and timing of wild stocks in the Discovery region (Stewart Johnson, personal communication), such an analysis may be possible in the future. But it is beyond the scope of the present study. Finally, during a disease outbreak such as in 2001, multiple farms can be infected and shedding and this can obviously have a cumulative effect. That is, a farm for which the minimum infective threshold is not exceeded if only one upstream farm is infected might however become infected if two or more farms upstream are infected. Though our connectivity matrices do not account for these cumulative effects, the particle tracking output used in their production does have the necessary information to compute them. Analogous to the sea lice concentration calculations described in [28], the total viral concentration at any location and any particular time is simply the sum of the concentrations arising from individual infected farms.

Sensitivity studies wherein the decay rate due to natural biota was reduced from 4.18 day^-1^ to 2.30 day^-1^ (see Fig 2 for the associated survival versus time curve) indicate that this parameter does alter the values in Fig 6. For example, with a disease outbreak at farm 16, the April risks of farm 17 becoming infected increase by 21% and 64% for the unvaccinated and vaccinated cases, respectively. And whereas before, the other farms had a zero risk of becoming infected, there are now small (< 1%) risks at farms 15, 18, and 3 for the unvaccinated case. This is to be expected as the virus cohort will travel further before its inactivation gets below the minimum infective dose threshold. We suspect that tests with the UV decay-versus-depth parameter would show similar sensitivities.

We also assumed that an infected farm sheds virus at a constant rate over an 18 day period. Though this is too long for present-day farm management practices (quarantine or depopulation would likely occur within a few days), the epidemics and shedding in 2001–02 were much longer [7]. And as discussed above, we have assumed that water-borne viruses only arise from a diseased farm whereas there is undoubtedly a temporally and spatially varying reservoir due to wild hosts that also contributes to naïve farm infections. We acknowledge that these assumptions, both individually and in combination, place potentially large uncertainties around the specific values in our connectivity matrix. Nevertheless, relative to one another, they should be fairly robust. Therefore, this study should provide some guidance for managing the relative risk of water-borne disease transmission and can be viewed as a baseline to which future refinements can be made as more observational and laboratory data become available.

## Appendix: Ocean Circulation Model Details

An application of the three-dimensional Finite Volume Community Ocean Model (FVCOM; [33–35]) has been developed for the Discovery Islands region and a simulation for April 1–28, 2010 has been evaluated against simultaneous observations from three current meter moorings and the harmonics computed from historical measurements at twenty-four tide gauges [10]. Though the model tidal elevations were found to have excellent agreement with the observations, profiles of model tidal speed versus depth generally did not capture observed vertical variations quite as well. However mean and sub-tidal flow fields were found to be reasonably accurate near the surface and near-surface model tidal current harmonics were shown to be in good agreement (see [10] for details) with those used to produce annual predictions at five sites in [9]. As salmon net-pen sites in British Columbia rarely exceed an 18m depth, it was felt that this agreement was sufficiently accurate for the viral transport estimates to be computed here. Until high resolution atmospheric and hydrological models are also developed for this region, uncertainties associated with the wind and freshwater forcing fields [36] will carry over to uncertainties in the ocean circulation fields. So there are limitations on further accuracy improvements with the present model and forcing fields.

Since the publication of [10], simulations have been repeated for April 1–28 and then extended from April 29 to October 31, 2010. Although the April 1–28, 2010 hindcast ignored heat flux (surface water temperature) forcing on the grounds that it would have minimal impact at that time of the year (air and water temperatures were comparable and skies were generally overcast or raining [10]), this forcing is important later in the year (see Fig 7 in [10]) so it was included in an April re-run and the May-October simulations. There were also some minor changes to the grid (e.g., coastline or boundary nodes with only connections to two other boundary nodes were modified) and river discharge magnitudes, salinities and temperatures. As in [10], the April initial temperature and salinity conditions were computed from seasonal climatologies based on all available historical observations. The simulations were carried out in monthly segments with “hot-restarts” at the beginning of each May-October sub-run taken from the stored values at the end of the simulation for the preceding month. Discharges for the Homathko, Salmon, Campbell, and Oyster Rivers were taken from Water Survey of Canada observations (http://www.wateroffice.ec.gc.ca/google_map/google_map_e.html?search_by=p&province=BC) while those for the other eight rivers in the system were estimated from the relative size of their watersheds, as described in [10]. The associated discharge salinities and temperatures were assigned values based on mid-month climatological averages in the top 5 meters of the water column at grid nodes nearest the river mouths. Winds were computed from observations at the weather stations shown in Fig 1 of [10]. Heat fluxes for each of the short wave, long wave, sensible, and latent components were computed using standard bulk formulae [37] and observations from the weather stations (air temperature, solar radiation, humidity, wind) and the Barnes Bay farm (number 22 in Fig 1; water temperature).

**Fig 9:**
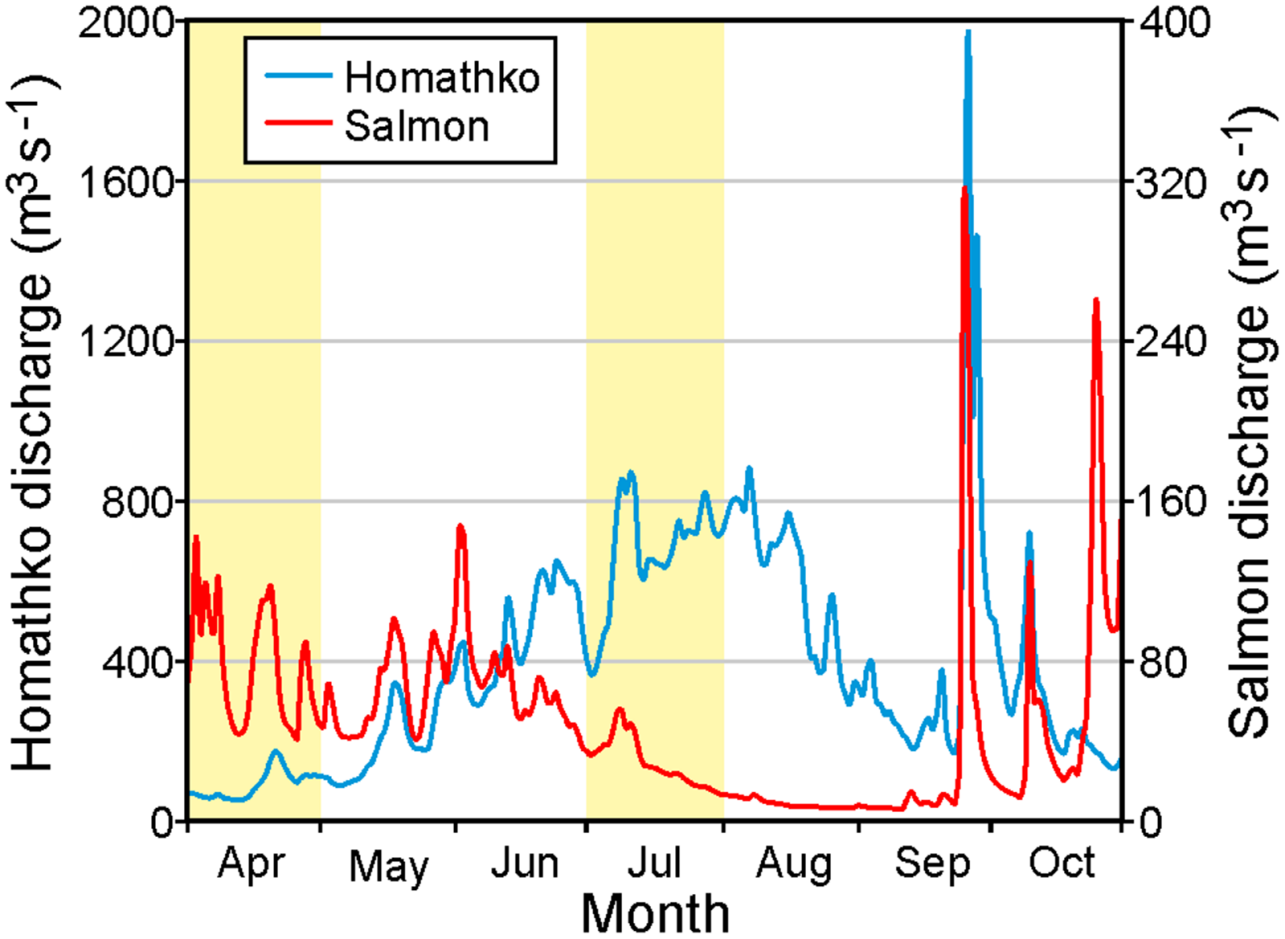
Homathko and Salmon River discharges (m^3^ s^-1^) for April-October 2010 with light shading denoting the months of April and July. Note the different vertical scaling.

Water-borne virus transport within the Discovery Islands will vary on time scales of hours to years. Tides, winds, and UV radiation [11] will affect ocean currents and IHNV inactivation at the shorter scales while inter-annual differences in the atmospheric conditions (e.g., air temperature, precipitation, short wave radiation), both directly and indirectly via their impact on the river discharges, mean that there will also be variations from year to year. However as the largest differences are expected to arise seasonally, viral transport simulations were conducted for April and July 2010 to capture typical spring and summer conditions. To illustrate the seasonal variations in freshwater input to the circulation model, Fig 9 shows the 2010 April-October daily discharges for the Homathko and Salmon Rivers (Fig 1), the two largest natural rivers in the region. Both rivers show longer term seasonal patterns superimposed on daily to weekly variations that are largely determined by the weather. The seasonal Homathko discharges are primarily driven by snow and glacial melt which typically ramp-up in mid-May and taper off in the fall. This means that the April discharges, and the estuarine oceanic currents that they generate, are generally much smaller than those in mid-summer. However there can be large storms in the late summer and early fall whose rainfall produces significant, but short duration, discharge events. On the other hand, the Salmon seasonal discharges are primarily driven by rainfall which peaks in the fall and winter and is generally much smaller in the summer. With the exception of the Campbell River whose flows are partially controlled by a dam (see [10]), the other Vancouver Island rivers generally follow the same pattern as the Salmon while the other BC mainland rivers follow the same pattern as the Homathko. This includes the Fraser River (the largest river in BC) that discharges into the Strait of Georgia to the south of our model domain and whose impact will be reflected in salinity and temperature conditions along the southern boundary of the model. Thus fresher water entering the Discovery region in the summer primarily comes from the mainland coast and southern boundary while that entering in the other seasons can come from the east, west, and south.

**Fig 10:**
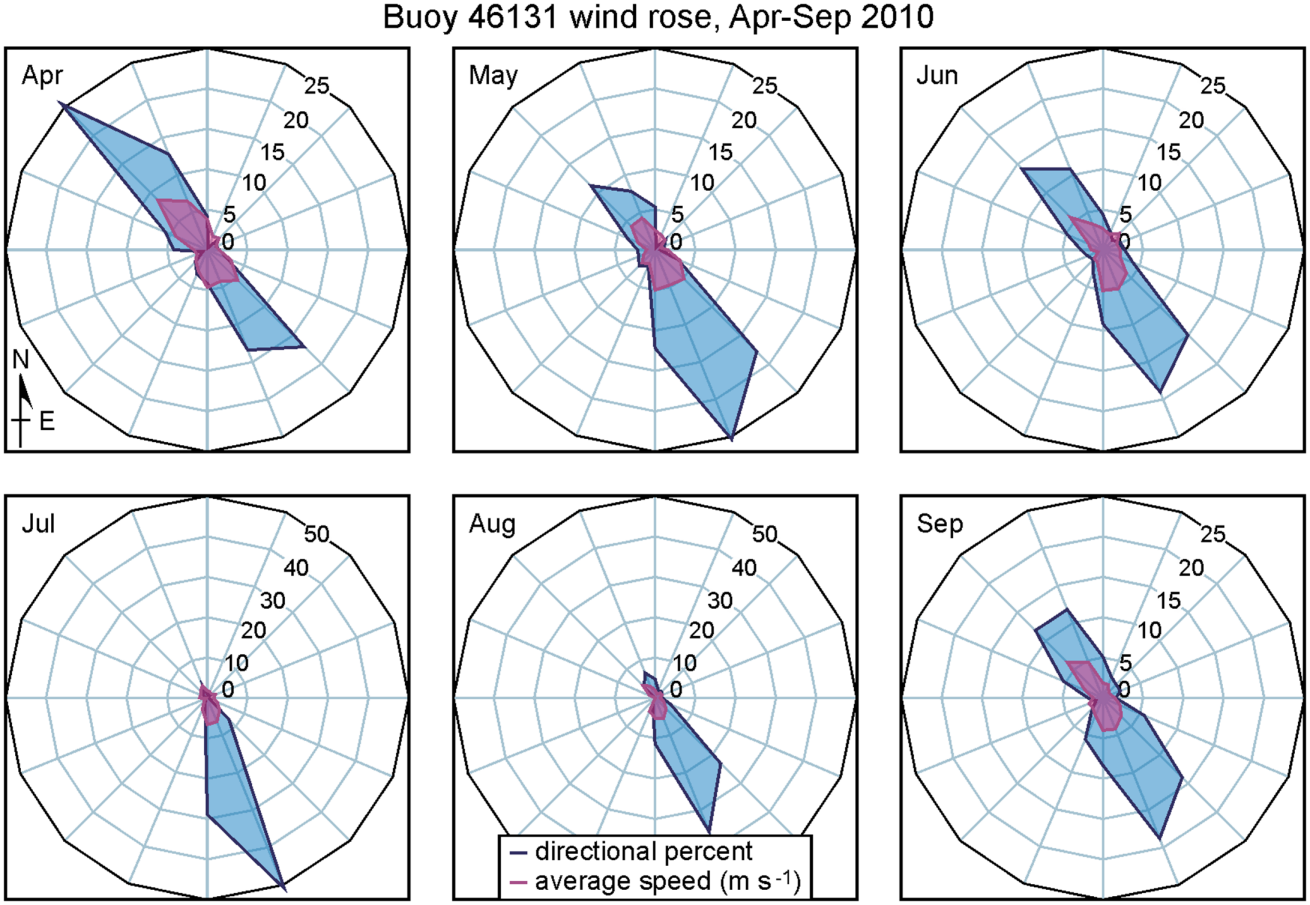
2010 monthly average wind roses at the Sentry Shoal weather station (Fig 1). Directional percent refers to the percentage of the time the wind was blowing toward the direction shown (±11.25°), while average speed (m s^-1^) refers to the average magnitude of the wind in each of those directions. Note the different axis scales for July and August.

Fig 10 shows 2010 monthly wind roses based on hourly observations at the Environment Canada Sentry Shoal weather station (Fig 1). Winds are predominantly from, or toward, the northwest. Relatively equal percentages in each direction in April are seen to shift to largely from the northwest in July and August and then back to both directions in September. (The October wind rose is similar to that for April.) Average wind speeds are seen to be generally comparable for all the months. Though strong topographic steering means that winds in the other channels and fiords of the Discovery Islands region will vary from those at Sentry Shoal (see Fig 6 in [10]), they generally exhibit similar seasonal variations.

**Fig 11:**
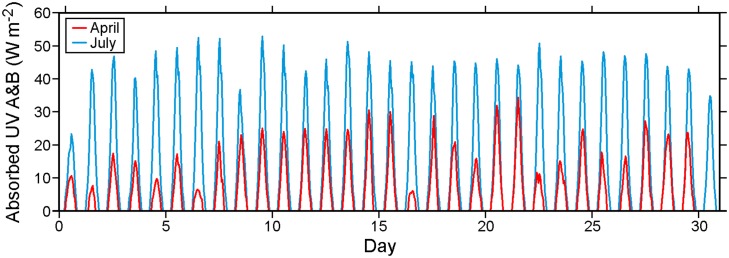
Average hourly UV radiation (A and B, W m^-2^) absorbed by the ocean for April and July 2010.

Fig 11 shows average hourly UV radiation absorbed at the ocean surface for April and July 2010. Values are restricted to the A and B frequency bands, as measured at the DFO weather stations (Fig 6 in [10]), and have been reduced by 8%, the average ocean surface albedo for UV. Zero values during the night are clearly evident, and the longer days, less cloud cover and a higher solar angle mean considerably more radiation entering surface waters in July than in April.

Based on the river discharges and winds shown in Figs 9 and 10, we expect that most of May and June should have wind- and buoyancy-driven currents (and thus virus dispersion patterns) similar to those for April while August should have currents similar to those for July. Though the lunar elliptical orbit around the earth means that tidal currents will exhibit a small variation on time scales longer than one month (e.g., perigean spring tides), on shorter time scales tidal current magnitudes and timings should remain relatively unchanged over the five month period. So the evaluation of model tidal currents (and elevations) carried out for April in [10] applies here. Though our ocean circulation and subsequent virus tracking simulations only cover 2010, similar seasonal patterns in the forcing fields (Figs 9, 10 and 11) generally exist in, and thus the model results should also apply to, other years.

**Fig 12:**
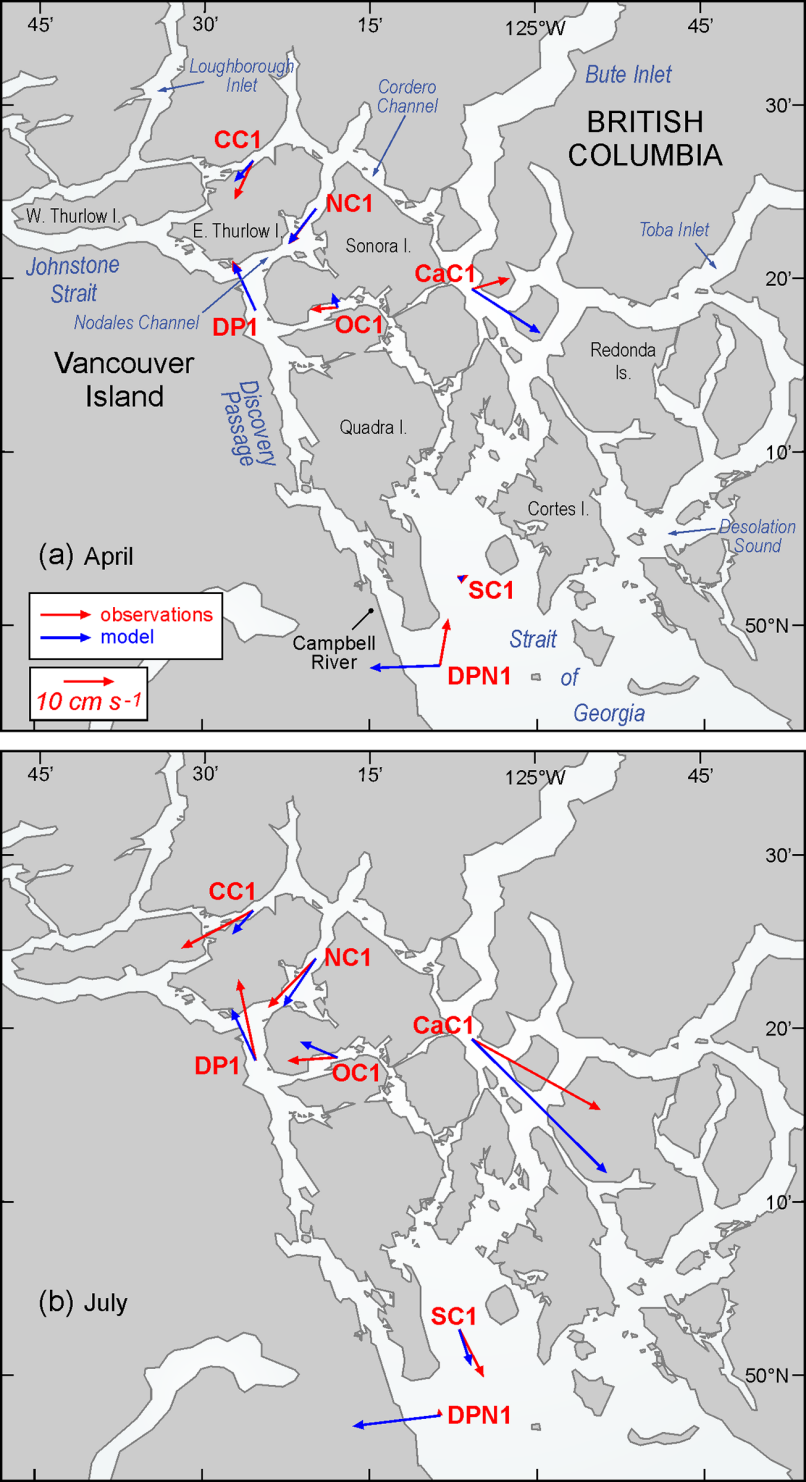
Monthly-averaged observed (red) and model blue) near surface currents for a) April and b) July, as listed and described in Table 1.

Table 1 and Fig 12 compare monthly-averaged, near surface, model currents for April and July 2010 against those observed in analogous months (but not necessarily 2010) at seven ADCP moorings (Fig 1) in the Discovery region. As the observations spanned 2010 to 2013, some of the differences are undoubtedly due to inter-annual variability. The agreement at sites DP1 and NC1 is very good and with the exception of DPN1, it is reasonable at the others. Differing directions in cases such as the April currents at OC1 and CaC1 are due to small scale eddies that have either not been accurately captured, or have been overly represented, by the model. (Examples are seen in Figs 7 and 8.)

**Table 1:** April and July monthly-averaged velocities (cm s^-1^, degrees counter-clockwise from east) over the top 20m (or portion thereof) at each of the ADCP locations (Fig 1).

1	2	3	4	5	6
site	Month, year	ADCP depth range (m) (and bin spacing) or top bin depth	Model average velocity for depth range or depth in column 3 (speed, dir.)	ADCP average velocity for depth range or depth in column 3 (speed, dir)	ADCP non-tidal RMS velocity for depth range or depth in column 3 (u,v)
DP1	April 2010 April 2011	21.7 16.6–21.6 (5.0)	11.3, 115 10.5, 115	10.2, 130 11.1, 115	(9,10) (9,10)
DP1	July 2010	19.6–24.6 (5.0)	11.4, 115	16.6, 102	(13,18)
NC1	April 2010	4.0–21.0 (5.0)	9.0, 232	8.9, 233	(4,5)
NC1	July 2010	17.2–22.2 (4.0)	11.6, 236	13.7, 226	(5,7)
DPN1	April 2010	26.7	14.1, 182	9.5, 80	(35,24)
DPN1	July 2010	8.3–24.3 (8.0)	17.6, 187	1.3, 99	(23,18)
OC1	April 2011 April 2012	3.7–19.7 (2.0) 5.9–21.9 (4.0)	3.1, 109 3.0, 115	5.4, 184 2.8, 167	(6,5) (6,5)
OC1	July 2011	5.9–21.9 (4.0)	8.1, 157	10.0, 184	(6,4)
CC1	April 2012	8.5–23.5 (5.0)	5.6, 229	8.6, 245	(6,7)
CC1	July 2011	8.5–23.5 (5.0)	6.3, 229	16.2, 208	(7,6)
SC1	April 2013	5.0–21.0 (2.0)	1.3, 43	2.0, 27	(7,9)
SC1	July 2012	4.0–20.0 (4.0)	7.6, 288	10.7, 297	(9,8)
CaC1	April 2013	6.1–22.1 (4.0)	16.3, 327	7.8, 16	(8,7)
CaC1	July 2012	6.1–22.1 (4.0)	38.2, 315	29.5, 331	(9,8)

In cases where the upward looking ADCP coverage did not extend to the top 20m, values for the top bin are given instead. The last column shows observed root mean square speeds (cm/s) in the (u = east-west, v = north-south) directions after the tides and mean flow have been removed.

As DPN1 is not near any of the salmon farms, inaccuracies in its mean model currents should not significantly affect our viral dispersion results. Nevertheless, we are investigating the source of these inaccuracies (which were also noted in [10]) and feel they arise from a combination of the following three reasons. The first is the proximity of this site to the southern boundary of the model and its susceptibility to the relatively simple nudging-to-climatology conditions that are imposed there. These conditions will be replaced with a time series of values from a model covering a larger domain (e.g., [38]) in the next round of model improvements and that should help the accuracy of the currents in the northern Strait of Georgia and in particular, at DPN1. The second reason is that the DPN1 observations themselves are not as accurate as those at the other sites as the mooring regularly exhibited tilts larger than 20° from the vertical. This indicates that it was being dragged down in the water column by strong currents, which in this case, arose from the passing of a southward eddy that separates from Cape Mudge (Fig 1) as the flood tide enters the northern Strait of Georgia. The accuracy to which this eddy has been captured by the model is the third contributor to the large discrepancies at DPN1. The ADCP observations show a spike of near surface currents that can exceed 2 m s^-1^ when the eddy passes while the model has a smaller spike that lasts longer in time. These differences impact both the tidal constituent and mean-flow harmonics arising from analyses of the model and observational time series [39] and thus contribute to the differences seen in Table 1 and Fig 12. Though attempts to reconcile these differences via the acquisition of satellite images and model sensitivity tests are underway, this work is beyond the scope of the present study and will not be reported here.

Note that with the exception of DPN1, the observed July average currents are stronger than those in April. Though the change in wind direction (Fig 10) may play a minor role, this increase is largely due to more freshwater discharge entering the system from the BC mainland rivers (Fig 9). For example, differences between the April and July observed mean flows at mooring CaC1 are largely a consequence of increased freshwater inputs to both Bute and Toba Inlets. (From Fig 9, the average Homathko discharges in April and July are 90.5 and 665 m^3^ s^-1^, respectively.) The model currents show a similar April-to-July increase, though generally not by as much as seen in the observations. The final column in Table 1 gives the root mean square speeds (cm s^-1^) in each the east-west and north-south directions after the mean flows (4^th^ column) and tides have been removed. (The tidal analysis [39] used exactly same tidal constituents and inference parameters to de-tide the time series at each site.) They can be interpreted as a proxy for dispersion due to variations in the wind and freshwater discharge. Note that in most, but not all, locations they are larger in July. Thus in the absence of UV radiation effects, we would expect viral infectious units to be transported further and undergo more dispersion in July than in April. However, stronger UV values in July may be sufficient to negate this infection potential and lower the risk of greater viral dispersal.

## References

[1] Murray AG (2013) Epidemiology of the spread of viral diseases under aquaculture. Current Opinion in Virology, 3:74–78 10.1016/j.coviro.2012.11.002 23206337

[2] McVicar AH (1997) Interaction of pathogens in aquaculture with wild fish populations. Bulletin of the European Association of Fish Pathologists, 17(6), 197–200.

[3] Kurath G, and Winton JR. (2011) Complex dynamics at the interface between wild and domestic viruses of finfish. Current Opinions in Virology. 1:73–80. 10.1016/j.coviro.2011.05.010 22440571

[4] Saksida SM, Gardner I, and Kent ML. (2014) Sea lice management on salmon farms in British Columbia, Canada In Salmon Lice: An Integrated Approach to Understanding Parasite Abundance and Distribution. ed. Jones S, Beamish R, pp. 235–78. Chichester, UK: Wiley-Blackwell.

[5] Johansen LH, Jensen I, Mikkelsen H, Bjørn PA, Jansen PA, and Bergh O. (2011) Disease interaction and pathogens exchange between wild and farmed fish populations with special reference to Norway. Aquaculture 315, 167–186. 10.1016/j.aquaculture.2011.02.014

[6] St-Hilaire S, Ribble CS, Stephen C, Anderson E, Kurath G, and Kent ML. (2002) Epidemiological investigation of infectious hematopoietic necrosis virus in salt water net-pen reared Atlantic salmon in British Columbia, Canada. Aquaculture 212: 49–67.

[7] Saksida SM. (2006) Infectious haematopoietic necrosis epidemic (2001 to 2003) in farmed Atlantic salmon Salmo salar in British Columbia. Dis. Aquat. Organ. 72(3): 213–23. 17190200 10.3354/dao072213

[8] Lafferty KD, Harvel DC, Conrad JM, Friedman CS, Kent ML, Kuris AM, et al (2014) Infectious diseases affect marine fisheries and aquaculture economics. Annu Rev Mar Sci 2015 7:11.1–12.26. 10.1146/annurev-marine-010814-015646 25251276

[9] Tide Canadian and Tables Current (2010) Discovery Passage and the West Coast of Vancouver Island, Volume 6 Fisheries and Oceans Canada, Ottawa, 131 pp.

[10] Foreman MGG, Stucchi DJ, Garver KA, Tuele D, Isaac J, Grime T, et al (2012) A circulation model for the Discovery Islands, British Columbia. Atmosphere-Ocean 50(3): 301–316.

[11] Garver KA, Mahony-Grant AM, Stucchi D, Richard J, Van Woensel C, and Foreman M. (2013) Estimation of Parameters Influencing Waterborne Transmission of Infectious Hematopoietic Necrosis Virus (IHNV) in Atlantic Salmon (*Salmo salar*). PLoS ONE 8(12): e82296 10.1371/journal.pone.0082296 24340016 PMC3855332

[12] Salama NKG, and Rabe B. (2013) Developing models for investigating the environmental transmission of disease-agents with open-cage salmon aquaculture, Aquaculture Environment Interactions, 4: 91–115.

[13] Stene A, Viljugrein H, Yndestad H, Tavornpanich S, and Skjerve E. (2013) Transmission dynamics of pancreas disease (PD) in a Norwegian fjord: aspects of water transport, contact networks and infection pressure among salmon farms, Journal of Fish Disease, 10.111/jfd.12090 23452114

[14] Viljugrein H, Staalstrøm A, Molvaer J, Urke HA, and Jensen PA. (2009) Integration of hydrodynamics into a statistical model on the spread of pancreas disease (PD) in salmon farming, Diseases of Aquatic Organisms, 88: 35–44, 10.3354/dao02151 20183963

[15] Asplin L, Boxaspen KK, and Sandvik AD. (2011) Modelling the distribution and abundance of planktonic larval stages of *Lepeophtheirus salmonis* in Norway in *Salmon Lice*: *An integrated approach to understanding parasite abundance and distribution*. 10.1002/9780470961568 Wiley-Blackwell, Oxford, p 31–50.

[16] Mardones FO, Perez AM, and Carpenter TE. (2009) Epidemiologic investigation of the re-emergence of infectious salmon anemia virus in Chile, Diseases of Aquatic Organisms, Vol. 84: 105–114, 10.3354/dao02040 19476280

[17] Olivares GR, Sepulveda HH, and Yannicelli B. (2014) Definition of sanitary boundaries to prevent ISAv spread between salmon farms in Southern Chile based on numerical simulations of currents. Submitted to Estuarine, Coastal and Shelf Science.

[18] Murray AG, Amundrud TL, Penston MJ, Pert CC, and Middlemas SJ. (2011) Abundance and distribution of larval sea lice in Scottish coastal waters, in *Salmon Lice*: *An integrated approach to understanding parasite abundance and distribution* *In*: Jones S. and Beamish RJ. (*eds*). Salmon Lice: An Integrated Approach to Understanding Parasite Abundance and Distribution. 10.1002/9780470961568.ch4, Wiley-Blackwell, Oxford.

[19] Gillibrand PA and Willis KJ. (2007) Dispersal of sea louse larvae from salmon farms: modelling the influence of environmental conditions and larval behaviour. Aquatic Biology. 1, 63–75.

[20] Amundrud TL and Murray AG. (2009) Modelling sea lice dispersion under varying environmental forcing in a Scottish sea loch. Journal of Fish Diseases. 32(1):27–44. 10.1111/j.1365-2761.2008.00980.x 19245629

[21] Adams T, Black K, MacIntyre C, MacIntyre I, and Dean R. (2012) Connectivity modelling and network analysis of sea lice infection in Loch Fyne, west coast of Scotland. Aquaculture Environment Interactions, 3: 51–63, 10.3354/aei00052

[22] Stagg RM. (2003) The eradication of an outbreak of clinical infectious salmon anaemia from Scotland In: Miller O, Cipriano RC (eds.) International response to infectious salmon anemia: prevention control, and eradication: Proc Symp 3–4 Sept 2002, New Orleans, LA Tech Bull No 1902. US Dept Agric, Anim Plant Health Inspect Serv, US Dept Interior, US Geol Surv, US Dept Commerce, Natl Mar Fish Serv, Washington, DC, p 111–124.

[23] Murray AG, Amundrud TL, and Gillibrand PA. (2005) Models of hydrodynamic pathogen dispersal affecting Scottish salmon production: modelling shows how Scotland eradicated ISA, but not IPN. Bull Aquacult Assoc Can 105: 79–86.

[24] Greenberg D., Shore JA, Page FH, and Dowd M. (2005) A finite element circulation model for embayments with drying intertidal areas and its application to the Quoddy Region of the Bay of Fundy. Ocean Model. 10, 211–231.

[25] Page FH, Chang B, Losier RJ, Greenberg DA, Chaffey JD, and McCurdy EP. (2005) Water circulation and and management of infectious salmon anemia in the salmon aquaculture industry of southern Grand Manan Island, Bay of Fundy. *Canadian Technical Report of Fisheries and Aquatic Sciences*, 2595: iii+78 p.

[26] Chang BD, Page FH, Losier RJ, Greenberg DA, Chaffey JD, and McCurdy EP. (2005) Water circulation and management of infectious salmon anemia in the salmon aquaculture industry of Cobscook Bay, Maine and adjacent southwestern New Brunswick. *Canadian Technical Report of Fisheries and Aquatic Sciences*, 2598: iii+54 p.

[27] Gustafson LL, Ellis SK, Beattie MJ, Chang BD, Dickey DA, Robinson TL, et al (2007) Hydrographics and the timing of infectious salmon anemia outbreaks among Atlantic salmon (Salmo salar L.) farms in the Quoddy region of Maine, USA and New Brunswick, Canada. Preventive VeterinaryMedicine 78, 35–56. 10.1016/j.prevetmed.2006.09.006 17097172

[28] Stucchi D, Guo M, Foreman MGG, Czajko P, Galbraith M, Mackas DM, et al (2011) Modelling sea lice production and concentrations in the Broughton Archipelago, British Columbia, in *Salmon Lice*: *An integrated approach to understanding parasite abundance and distribution* *In*: Jones S. and Beamish R. J. (*eds*). Salmon Lice: An Integrated Approach to Understanding Parasite Abundance and Distribution. 10.1002/9780470961568.ch4, Wiley-Blackwell, Oxford.

[29] Salonius K, Simard N, Harland R, and Ulmer JB. (2007). The road to licensure of a DNA vaccine. Current Opinion in Investigational Drugs 8(8):635–641. 17668365

[30] Cohen BI. (2012) The uncertain future of Fraser River sockeye: Commission of the Inquiry into the Decline of the Sockeye Salmon in the Fraser River, Public Works and Government Services Canada, Ottawa Available: www.cohencommission.ca.

[31] Kurath G., Garver KA, Troyer RM, Emmenegger EJ, Einer-Jensen K, and Anderson ED. (2003). Phylogeography of infectious hematopoietic necrosis virus in North America. Journal of General Virology 84:803–814. 12655081 10.1099/vir.0.18771-0

[32] Traxler GS, Roome JR, and Kent ML. (1993). Transmission of infectious hematopoietic necrosis virus in seawater. Diseases of Aquatic Organisms Vol. 16:111–114).

[33] Chen C, Liu H, and Beardsley RC. (2003) An unstructured, finite-volume, three-dimensional, primitive equation ocean model: application to coastal ocean and estuaries, Journal of Atmospheric and Oceanic Technology. 20, 159–186.

[34] Chen C, Haung H, Beardsley RC, Xu Q, Limeburner R, Cowles GW, et al (2011) Tidal dynamics in the Gulf of Maine and New England Shelf: An application of FVCOM. *J*. *Geophys*. *Res*., 116 C12010, 10.1029/2011JC007054

[35] Tian R, Chen C, Qi J, Ji R, Beardsley RC, and Davis C. (2014) Model study of nutrient and phytoplankton dynamics in the Gulf of Maine: patterns and drivers for seasonal and interannual variability. *ICES Journal of Marine Science*. 10.1093/icesjms/fsu090

[36] Morrison J, Foreman MGG, and Masson D. (2011) A Method for Estimating Monthly Freshwater Discharge Affecting British Columbia Coastal Waters. Atmosphere-Ocean: 10.1080/07055900.2011.637667

[37] Fairall CW, Bradley EF, Hare JE, Grachev AA, and Edson JB. (2003) Bulk parameterization of air-sea fluxes: Updates and verification for the COARE algorithm, J. Clim., 16, 571–591, 10.1175/1520-0442(2003)016<0571:BPOASF>2.0.CO;2

[38] Masson D, and Fine I. (2012) Modeling seasonal to inter-annual ocean variability of coastal British Columbia, Journal of Geophysical Research, 117, C10019, doi: 1029/2012JC008151

[39] Foreman MGG, Cherniawsky JY, and Ballantyne VA. (2009) Versatile harmonic tidal analysis: improvements and applications. Journal of Atmospheric and Oceanic Technology, 10.1175/2008JTECHO615.1, 26: 806–817.

